# Salivary Protein Profiles among HER2/neu-Receptor-Positive and -Negative Breast Cancer Patients: Support for Using Salivary Protein Profiles for Modeling Breast Cancer Progression

**DOI:** 10.1155/2012/413256

**Published:** 2012-04-10

**Authors:** Charles F. Streckfus, Daniel Arreola, Cynthia Edwards, Lenora Bigler

**Affiliations:** Department of Diagnostic Sciences, UTHSC, Dental Branch, Behavioral and Biomedical Sciences Building, Rm. 5322, Houston, TX 77054, USA

## Abstract

*Purpose*. The objective of this study was to compare the salivary protein profiles from individuals diagnosed with breast cancer that were either HER2/neu receptor positive or negative. *Methods*. Two pooled saliva specimens underwent proteomic analysis. One pooled specimen was from women diagnosed with stage IIa HER2/neu-receptor-positive breast cancer patients (*n* = 10) and the other was from women diagnosed with stage IIa HER2/neu-receptor-negative cancer patients (*n* = 10). The pooled samples were trypsinized and the peptides labeled with iTRAQ reagent. Specimens were analyzed using an LC-MS/MS mass spectrometer. *Results*. The results yielded approximately 71 differentially expressed proteins in the saliva specimens. There were 34 upregulated proteins and 37 downregulated proteins.

## 1. Introduction

Clinicopathologic factors such as histological type, tumor size, tumor grade, hormone receptor status, lymph node involvement, and HER-2/neu overexpression are recognized as having prognostic use in breast cancer management. HER-2/neu (HER2), also known as c-*erb*B-2, is a biomarker assayed in tissue biopsies from women diagnosed with malignant breast tumors [1, 2]. Used primarily as a prognostic indicator, HER2/neu protein is overexpressed in approximately 20%–30% of malignant breast tumors and has been used in postoperative followup evaluation as an indicator of patient relapse [3–6].

The evolution of HER2 testing, first as a prognostic marker assay and later as a diagnostic test to determine eligibility for trastuzumab-targeted therapy, has expanded the role of traditional diagnostic pathology. Unlike most testing performed by anatomic pathologists, which serves as an adjunct to establishing a diagnosis, the results of HER2 testing stand alone in determining which patients are likely to respond to trastuzumab therapy. HER2 status may also predict sensitivity to certain cytotoxic drugs and antiestrogens [3–6].

Currently, two testing methods are approved by the Food and Drug Administration (FDA) for HER2 assessment in the laboratory [1, 2]. They are immunohistochemical analysis (IHC) and fluorescence *in situ* hybridization (FISH). Commercially available, FDA-approved HER2 assays are available for both methods. Immunohistochemical analysis and FISH have the advantage over other assay methods (i.e., those requiring homogenization) because they are morphologically driven. This allows for the direct evaluation of tumor cells, correlation with other morphologic features, and the ability to assay smaller patient samples such as needle core biopsy specimens [1, 2].

While clinical treatment choices are critical, the actual tests used to determine HER2 status have demonstrated a number of pitfalls. One such pitfall is the number of false negatives and false positives associated with the tests. This creates a treatment dilemma as it can result in situations where patients requiring trastuzumab-targeted therapy may not receive it, while those receiving trastuzumab-targeted therapy should not receive it [6–8].

Not everyone is convinced that the problem is as simple as false negatives and false positives. One problem may be with the cutoff points that scientists have established to delineate between HER2 negative and positive in IHC testing. Data from several preclinical and clinical studies suggest that trastuzumab activity does not strictly require HER2 overexpression or gene amplification, as is currently thought. Instead, even tumor cells that express a lower level of the protein might respond to a trastuzumab-chemotherapy combination. Currently, tumors that have moderate amounts of HER2 protein would be scored as 1+ or 2+ on IHC tests and would be called negative because they are below the predetermined cutoff value for the test [1–8].

Even FISH testing, which is considered the “gold standard” for detecting gene amplification, has problems. The test measures the ratio between the area surrounding the HER2 gene on chromosome 17 and other parts of chromosome 17. That means a cell that has extra copies of chromosome 17, called polysomy 17, appears negative by FISH but actually has extra copies of HER2, and probably expresses more protein than a wild-type cell would [1, 2].

The inconsistency in the test may stem, in part, from heterogeneity of HER2 expression in the tumor sample. If so, then multiple tests on the same tumor could yield different results, even though the tests are working as designed. The testing problem has been a matter of discussion, and how it will be resolved remains unclear. However, clinicians and researchers agree that current technology to assess HER2 status needs improvement [1–8].

Therefore, the purpose of this study was twofold. The first objective was to compare salivary protein profiles among Her2-receptor-positive and -negative breast cancer patients and secondly, to support the theory for using salivary protein profile expression as a method for modeling breast cancer progression [9, 10].

## 2. Methods

### 2.1. Design

The investigators protein profiled three pooled, stimulated whole saliva specimens. One specimen consisted of pooled saliva from 10 stage IIa (T_2_N_0_M_0_) HER2-receptor-status-positive invasive ductal carcinoma patients (IDC), and the second pooled specimen was from 10 subjects diagnosed with stage IIb (T_2_N_0_M_0_), HER2-receptor-status-positive invasive ductal carcinoma. The cancer cohorts were estrogen, progesterone receptor status negative as determined by the pathology report. Histological grade was not available for this study. The subjects were matched for age and race and were nontobacco users.

The participating subjects were given an explanation about their participation rights, and they signed an IRB consent form. The saliva specimens and related patient data are nonlinked and bar-coded in order to protect patient confidentiality. This study was performed under the UTHSC IRB-approved protocol number HSC-DB-05-0394. All procedures were in accordance with the ethical standards of the UTHSC IRB and with the Helsinki Declaration of 1975, as revised in 1983.

### 2.2. Saliva Collection and Sample Preparation

Stimulated whole salivary gland secretion is based on the reflex response occurring during the mastication of a bolus of food. Usually, a standardized bolus (1 gram) of paraffin or a gum base (generously provided by the Wrigley Co., Peoria, IL) is given to the subject to chew at a regular rate. The individual, upon sufficient accumulation of saliva in the oral cavity, expectorates periodically into a preweighed disposable plastic cup. This procedure is continued for a period of five minutes. The volume and flow rate is then recorded along with a brief description of the specimen's physical appearance [9, 10]. The cup with the saliva specimen is reweighed and the flow rate determined gravimetrically. A protease inhibitor from Sigma Co. (St. Louis, MI, USA) is added along with enough orthovanadate from a 100 mM stock solution to bring its concentration to 1 mM. The treated samples were centrifuged for 10 minutes at top speed in a tabletop centrifuge. The supernatant was divided into 1 mL aliquots and frozen at −80.

### 2.3. Two-Dimensional Gel Analysis (2D DIGE)

#### 2.3.1. Sample Preparation

2D DIGE and protein ID was performed by Applied Biomics, Inc. (Hayward, CA). Proteins from the saliva were precipitated by methanol then resuspended in a 2D cell lysis buffer (30 mM Tris-HCl, pH 8.8, containing 7 M urea, 2 M thiourea, and 4% CHAPS). Protein concentration was measured using Bio-Rad protein assay method.

#### 2.3.2. CyDye Labeling

For each sample, 30 ug of protein was mixed with 1.0 *μ*L of diluted CyDye and kept in dark on ice for 30 min. Samples from each pair were labeled with Cy2 and Cy5, respectively. The labeling reaction was stopped by adding 1.0 *μ*L of 10 mM Lysine to each sample, and incubating in dark on ice for additional 15 min. The labeled samples were then mixed together. The 2X 2D sample buffer (8 M urea, 4% CHAPS, 20 mg/mL DTT, 2% pharmalytes, and trace amount of bromophenol blue), 100 *μ*L destreak solution, and Rehydration buffer (7 M urea, 2 M thiourea, 4% CHAPS, 20 mg/mL DTT, 1% pharmalytes, and trace amount of bromophenol blue) were added to the labeling mix to make the total volume of 250 *μ*L. They were Mixed well and spined before loading the labeled samples into strip holder.

#### 2.3.3. IEF and SDS-PAGE

After loading the labeled samples, IEF (pH3-10 Linear) was run following the protocol provided by Amersham BioSciences. Upon finishing the IEF, the IPG strips were incubated in the freshly made equilibration buffer 1 (50 mM Tris-HCl, pH 8.8, containing 6 M urea, 30% glycerol, 2% SDS, trace amount of bromophenol blue, and 10 mg/mL DTT) for 15 minutes with gentle shaking. Then the strips were rinsed in the freshly made equilibration buffer 2 (50 mM Tris-HCl, pH 8.8, containing 6 M urea, 30% glycerol, 2% SDS, trace amount of bromophenol blue, and 45 mg/mL DTT) for 10 minutes with gentle shaking. Next, the IPG strips were rinsed in the SDS-gel running buffer before transferring into 13.5% SDS gels. The SDS gels were run at 15°C until the dye front exuded out of the gels.

#### 2.3.4. Image Scan and Data Analysis

Gel images were scanned immediately following the SDS-PAGE using Typhoon TRIO (Amersham BioSciences). The scanned images were then analyzed by Image Quant software (version 6.0, Amersham BioSciences), followed by in-gel analysis using DeCyder software version 6.0 (Amersham BioSciences). The fold change of the protein expression levels was obtained from in-gel DeCyder analysis.

### 2.4. Top-Down Mass Spectrometry Using iTRAQ Labeling

A thorough explanation for the top-down mass spectrometry using iTRAQ labeling can be found in detail in previous publications [9, 10]. Briefly, the saliva samples were thawed and immediately centrifuged to remove insoluble materials. The supernatant was assayed for protein using the Bio-Rad protein assay (Hercules, CA, USA), and an aliquot containing 100 *μ*g of each specimen was precipitated with six volumes of −20°C acetone. The precipitate was resuspended and treated according to the manufacturer's instructions. Protein digestion and reaction with iTRAQ labels was carried out as previously described and according to the manufacturer's instructions (Applied Biosystems, Foster City, CA). Briefly, the acetone precipitable protein was centrifuged in a tabletop centrifuge at 15,000 ×g for 20 minutes. The acetone supernatant was removed and the pellet resuspended in 20 *Ų*L dissolution buffer. The soluble fraction was denatured, and disulfides were reduced by incubation in the presence of 0.1% SDS and 5 mM TCEP (tris-(2-carboxyethyl) phosphine) at 60°C for one hour. Cysteine residues were blocked by incubation at room temperature for 10 minutes with MMTS (methyl methane-thiosulfonate). Trypsin was added to the mixture to a protein: trypsin ratio of 10 : 1. The mixture was incubated overnight at 37°C. The protein digests were labeled by mixing with the appropriate iTRAQ reagent and incubating at room temperature for one hour. On completion of the labeling reaction, the four separate iTRAQ reaction mixtures were combined. Since there are a number of components that can interfere with the LCMSMS analysis, the labeled peptides are partially purified by a combination of strong cation exchange followed by reverse-phase chromatography on preparative columns. The combined peptide mixture is diluted 10-fold with loading buffer (10 mM, KH_2_PO_4_ in 25% acetonitrile at pH 3.0) and applied by syringe to an ICAT Cartridge-Cation Exchange column (Applied Biosystems, Foster City, CA) column that has been equilibrated with the same buffer. The column is washed with 1 mL loading buffer to remove contaminants. To improve the resolution of peptides during LCMSMS analysis, the peptide mixture is partially purified by elution from the cation exchange column in three fractions. Stepwise elution from the column is achieved with sequential 0.5 mL aliquots of 10 mM KH_2_PO_4_ at pH 3.0 in 25% acetonitrile containing 116 mM, 233 mM, and 350 mM KCl, respectively. The fractions are evaporated by Speed Vac to about 30% of their volume to remove the acetonitrile and then slowly applied to an Opti-Lynx Trap C18 100 *μ*L reverse-phase column (Alltech, Deerfield, IL) with a syringe. The column was washed with 1 mL of 2% acetonitrile in 0.1% formic acid and eluted in one fraction with 0.3 mL of 30% acetonitrile in 0.1% formic acid. The fractions were dried by lyophilization and resuspended in 10 *μ*L 0.1% formic acid in 20% acetonitrile solution. Each of the three fractions was analyzed by reverse-phase nano-LCMS/MS on an API QSTAR XL mass spectrometer (ABS Sciex Instruments).

### 2.5. Bioinformatics

The Swiss-Prot database was employed for protein identification, while the PathwayStudio bioinformatics software package was used to determine Venn diagrams that were also constructed using the NIH software program (http://ncrr.pnl.gov/). Graphic comparisons with log conversions and error bars for protein expression were produced using the ProQuant software. Candidates with either protein score C.I. percentage or Ion C.I. percentage greater than 95 were considered significant. 

## 3. Results

### 3.1. 2D Gel Results

**Figure 1:**
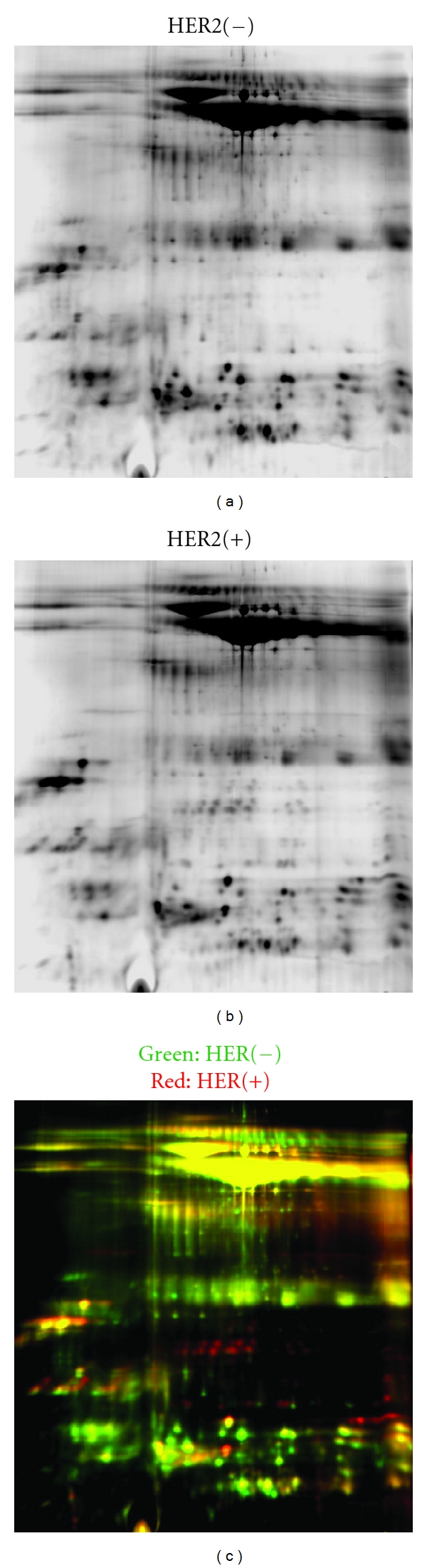
This figure illustrates the protein profiles for HER2/neu-receptor-positive and HER2/neu-receptor-negative samples. As shown in the far right red and green dyed gel comparisons, there are numerous differences between the two profiles.

**Figure 2:**
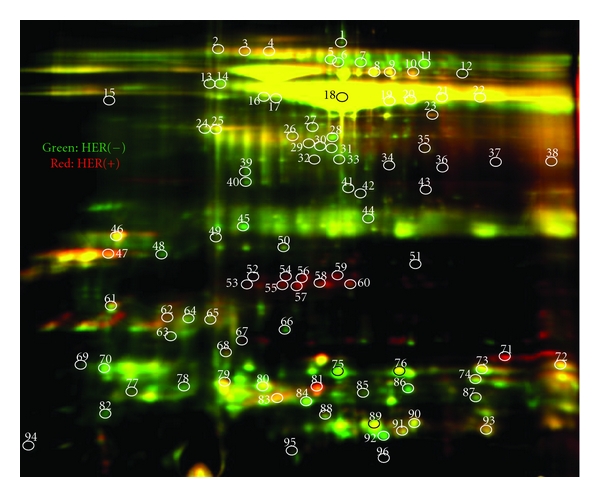
This figure demonstrates the differences in salivary protein profiles between HER2/neu -positive and HER2/neu -negative samples. Please change.

Figures 1 and 2 illustrate the results of the 2D gel analyses.  Figure 1 demonstrates the protein comparisons between the pooled HER2/neu-receptor-positive and the HER2/neu-receptor-negative pooled specimens.  Figure 2 represents the spots of interest that were selected up by Ettan Spot Picker (Amersham BioSciences) based on the in-gel analysis and spot picking design by DeCyder software. As shown, there are 96 spots of interest illustrated on the 2D gel analysis. This visually indicates the differing salivary protein patterns between HER2/neu-positive and HER2/neu-negative patients.

### 3.2. LC-MS/MS Mass Spectrometry Results

The results yielded 188 comparative salivary proteins among the HER2/neu-positive and HER2/neu-negative samples. Among the total number of proteins, 71 were significantly differentially expressed between the two specimens. There were 34 upregulated proteins and 37 downregulated proteins. Listed in Table 1 is the 34 upregulated proteins, and in Table 2, the 37 downregulated proteins.

Of the 34 proteins listed in Table 1, the mean percent peptide coverage for the complete panel of proteins was 50.3% (±19.6) with a range of 15.3% to 88.7% coverage. The mean protein ratio was 1.64  (±54.2) and ranged in value with a maximum of 3.13 to a maximum minimum of 1.12. Likewise, the mean alpha level was *P* < 0.007(±0.013) and ranged in value with a maximum of *P* < 0.04 to a maximum minimum of *P* < 0.000001.

**Figure 3:**
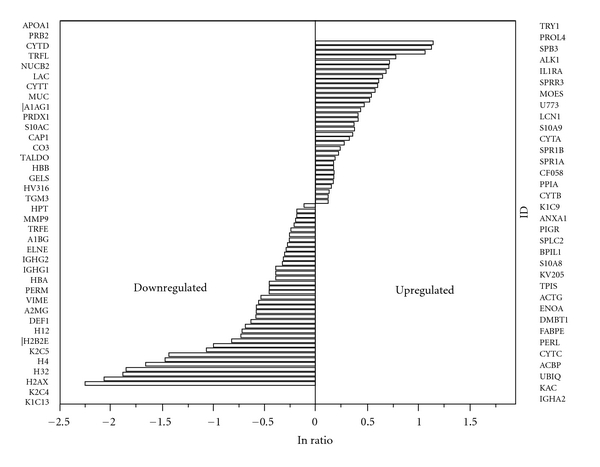
The figure represents the natural logarithm differential expression of salivary proteins. To the right and left of the figure listed in rank order of expression are the up- and downregulated proteins, respectively.

**Figure 4:**
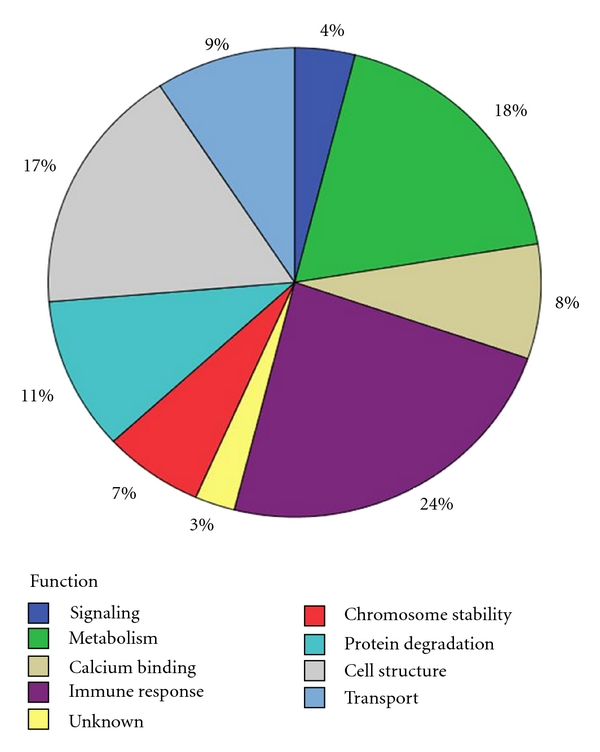
This figure represents the percentage of expressed proteins according to their cellular function.

Of the 37 downregulated salivary proteins listed in Table 2, the mean percent peptide coverage for the complete panel of proteins was 40.1% (±20.7) with a range of 9.7% to 100% coverage. The mean protein ratio was 1.64 (±0.561) and ranged in value with a maximum of 0.89 to a maximum minimum of 0.10. Likewise, the mean alpha level was *P* < 0.009  (±0.015) and ranged in value with a maximum of *P* < 0.05 to a maximum minimum of *P* < 0.000001.  Figure 3 illustrates the comparison of the log ratio of the relative intensity (HER2+/HER2−) of the total 71 up- and downregulated salivary proteins. Additionally, Figure 4 illustrates the protein function of the panel of 71 proteins. Proteins related to cellular metabolism and immune response comprised nearly 42% of the protein panel. Cellular structure constituted 17% of the protein panel, which is consistent with SKBR3 and other cell line protein analyses [15]. Additionally, there were a considerable number of histones that were present in the functional protein profile.

## 4. Discussion

To the best of our knowledge, this is the first attempt to determine salivary protein profile alterations related to HER2/neu receptor status. Therefore, we have only a few references by which to compare our data. Additionally, a complete proteomic catalog of the SKBR3 cell line is also not available to compare to the salivary protein profiles, which are altered secondary to HER2 receptor status. Of the articles that were identified through the PubMed Central (United States National Library of Medicine) search engine [11–17], twenty (28%) of the 71 of the salivary proteins were found cited among the manuscripts [11–17] and reported to be altered in the SKBR3 cell lines. These salivary proteins are listed in Table 3 with the references that cited the SKBR3 protein phenotype alterations.

**Figure 5:**
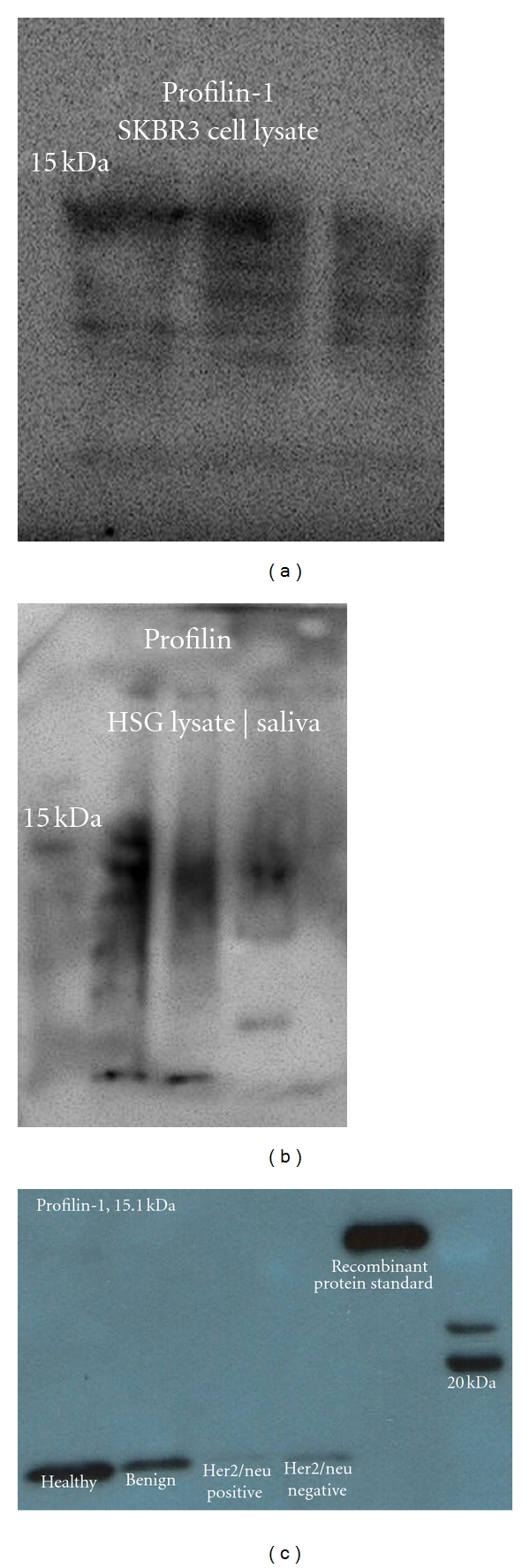
It illustrates the presence of profilin-1 in the SKBR3 and HSG cell lysates and saliva sampled from healthy, benign, and malignant tumor patients. Profilin is a downregulated protein in the presence of malignancy, and it is visualized by the lighter bands associated with malignancy. It is also worth noting that the Her2/neu-receptor-negative band is darker than the Her2/neu-receptor-positive counterpart suggesting further downregulation of the profiling-1 protein.

The numerous proteins in the panel need validation; however, the authors selected Profilin-1 as a test case to determine if the results of the proteomic analyses are feasible. As illustrated in Figure 5, Profilin-1 is present in both the SKBR3 and the human salivary gland (HSG) epithelial cell lines. Additionally, Profilin-1 is present in saliva and is downregulated to HER2-receptor-positive status. The pilot evidence supports the proteomic prediction.

It is worth noting that among the list (Table 3) of SKBR3 cell lysates proteins, there is a distinct absence of any proteins related to immune response to inflammatory cancer activity as compared to the salivary proteins listed in both Tables 1 and 2. The findings at this point suggest the strength of this *in vivo* model which could be indicative of response to therapy in the event these proteins are diminished in activity during treatment, thereby, indicating a response to therapy.

Further research is required to support the theories presented in this paper. For example, proteomic analyses of low-abundance proteins in the SKRB3 cell line lysates and saliva are required in order to address gaps in varying molecular pathways. Studies validating the panel of markers are also necessary, and an assessment of salivary protein modulation during trastuzumab therapy is essential.

## 5. Conclusions

 The results of the study suggest salivary protein alterations secondary to HER2 receptor status. This is not surprising considering that the ductal cells of the salivary glands contain HER2/neu receptors. More importantly, the study raises the notion that salivary gland protein secretions may be used as a “real-time”, *in vivo* model for studying breast cancer progression [9, 10]. Currently, there are three major methods for creating models for studying breast cancer progression [18]. The three methods utilize either breast cancer tumor cell lines xenografts of cell lines, and the third method uses animals—in this case genetically engineered mice [19] for creating various models for studying breast cancer [18]. All three models have generated useful insight into cancer progression; however, despite their utility, no individual model recapitulates all aspects of cancer progression [18, 19].

Hence, an adjunct, *in vivo* model system is needed for breast cancer tumorigenesis and predictive modeling for treatment response [18, 19]. The authors have demonstrated in previous studies that the salivary protein profiles are altered in the presence of ductal carcinoma *in situ *and are further altered in the presence of lymph node involvement [9, 10]. The preliminary findings of this paper coupled with previous studies do imply that this *in vivo* experimental model system, which utilizes one of the most easily obtained body fluids for marker analysis, may fill in the current gaps in our understanding of breast cancer pathogenesis, signaling pathways, the efficacy of varying chemotherapeutics, and identifying novel therapies. Most importantly, this new approach may shed new light on metastatic progression that is the principle cause of patient mortality.

## Figures and Tables

**Table 1:** Upregulated salivary proteins.

Access no.	Gene ID	%Cov	Name	Ratio	*P* value
P63261	ACTG	65.1	Actin, cytoplasmic 2	1.207	0.030
P07108	ACBP	28.7	Acyl-CoA-binding protein	1.161	0.002
P06733	ENOA	61.3	Alpha-enolase	1.193	0.030
P04083	ANXA1	56.1	Annexin A1	1.457	0.000
P03973	ALK1	36.4	Antileukoproteinase 1 precursor	2.188	0.004
Q8N4F0	BPIL1	27.3	Bactericidal/permeabilityincreasing	1.386	0.000
P35321	SPR1A	70.8	Cornifin-A	1.696	0.003
P22528	SPR1B	61.8	Cornifin-B	1.722	0.000
P01040	CYTA	87.8	Cystatin-A	1.788	0.000
P04080	CYTB	72.4	Cystatin-B	1.514	0.000
P01034	CYTC	62.3	Cystatin-C precursor	1.183	0.030
P35527	K1C9	25.5	Cytokeratin-9	1.512	0.001
Q9UGM3	DMBT1	39.0	Deleted in malignant brain tumors 1	1.191	0.000
Q01469	FABPE	45.2	Fatty acid-binding protein, epidermal	1.191	0.000
P01877	IGHA2	52.6	Ig alpha-2 chain C region	1.125	0.040
P01834	KAC	88.7	Ig kappa chain C region	1.128	0.040
P06309	KV205	31.6	Ig kappa chain V-II region GM607	1.274	0.030
P18510	IL1RA	31.6	Interleukin-1 receptor antagonist protein	2.050	0.000
P22079	PERL	46.5	Lactoperoxidase precursor	1.190	0.000
P31025	LCN1	46.6	Lipocalin-1 precursor	1.853	0.000
P26038	MOES	15.3	Moesin	1.991	0.020
P62937	PPIA	58.2	Peptidyl-prolyl cis-trans-isomerase A	1.553	0.003
P01833	PIGR	58.4	Polymeric-immunoglobulin receptor	1.452	0.000
Q16378	PROL4	58.2	Proline-rich protein 4 precursor	3.092	0.000
P05109	S10A8	58.1	Protein S100-A8	1.318	0.000
P06702	S10A9	57.0	Protein S100-A9	1.833	0.000
Q96DA0	U773	27.5	Protein UNQ773/PRO1567 precursor	1.930	0.000
P29508	SPB3	20.3	Serpin B3	2.910	0.000
Q96DR5	SPLC2	52.2	Short-palate lung and nasal epith. carc.	1.435	0.000
Q9UBC9	SPRR3	81.1	Small proline-rich protein 3	2.035	0.000
P60174	TPIS	42.2	Triosephosphate isomerase	1.257	0.012
P07477	TRY1	35.2	Trypsin-1 precursor	3.135	0.000
P62988	UBIQ	68.4	Ubiquitin	1.142	0.003
Q6P5S2	CF058	31.8	Uncharacterized protein C6orf58	1.603	0.000

**Table 2:** Downregulated salivary proteins.

Access no.	Gene ID	%Cov	Name	Ratio	*P* value
Q01518	CAP1	11.4	Adenylyl cyclase	0.7295	0.0077
P02763	|A1AG1	37.8	Alpha-1-acid glycoprotein	0.7571	0.0070
P04217	A1BG	21.2	Alpha-1B-glycoprotein	0.5602	0.0420
P01023	A2MG	23.3	Alpha-2-macroglobulin	0.4395	0.0000
P02647	APOA1	52.1	Apolipoprotein A-I	0.8928	0.0488
P02812	PRB2	100.0	Salivary prp 2	0.8307	0.0020
P01024	CO3	18.3	Complement C3	0.7227	0.0434
P28325	CYTD	39.4	Cystatin-D	0.8297	0.0027
P09228	CYTT	76.6	Cystatin-SA	0.7736	0.0003
P13646	K1C13	70.3	cytoskeletal 13	0.1058	0.0000
P19013	K2C4	72.3	cytoskeletal 4	0.1269	0.0000
P13647	K2C5	53.7	cytoskeletal 5	0.2291	0.0019
P06396	GELS	34.7	Gelsolin	0.6744	0.0004
P00738	HPT	51.2	Haptoglobin	0.6346	0.0000
P69905	HBA	31.7	Hemoglobin subunit alpha	0.5039	0.0000
P68871	HBB	56.5	Hemoglobin subunit beta	0.6774	0.0000
P16403	H12	18.8	Histone H1.2	0.3436	0.0056
P16104	H2AX	34.3	Histone H2A.x (H2a/x)	0.1531	0.0014
Q16778	|H2B2E	38.1	Histone H2B type 2-E	0.2383	0.0005
Q71DI3	H32	39.0	Histone H3.2	0.1580	0.0000
P62805	H4	50.5	Histone H4	0.1913	0.0000
P01857	IGHG1	46.4	Ig gamma-1 chain C region	0.5308	0.0000
P01859	IGHG2	42.0	Ig gamma-2 chain C region	0.5593	0.0000
P01777	HV316	20.2	Ig heavy chain V-III region TEI	0.6363	0.0408
P01842	LAC	83.8	Ig lambda chain C regions	0.7805	0.0017
P01871	MUC	30.2	Ig mu chain C region	0.7708	0.0009
P02788	TRFL	58.5	Lactotransferrin	0.8219	0.0012
P08246	ELNE	9.7	Leukocyte elastase	0.5594	0.0304
P14780	MMP9	28.0	Matrix metalloproteinase-9	0.5891	0.0002
P05164	PERM	37.0	Myeloperoxidase	0.4892	0.0016
P80303	NUCB2	27.1	Nucleobindin-2	0.8093	0.0370
Q06830	PRDX1	15.1	Peroxiredoxin-1	0.7488	0.0010
P07737	PROF1	65.7	Profilin-1	0.6752	0.0010
P80511	S10AC	40.2	Protein S100-A12	0.7363	0.0121
Q08188	TGM3	20.8	Glutamyltransferase E precursor	0.6357	0.0074
P02787	TRFE	51.6	Serotransferrin precursor	0.5730	0.0000
P37837	TALDO	29.7	Transaldolase	0.6777	0.0119
P08670	VIME	27.5	Vimentin	0.4832	0.0407

**Table 3:** Altered protein in saliva and in SKBR3 cell lines.

Access no.	Gene ID	Name	Reference
P06733	ENOA	Alpha-enolase	[11]
P04083	ANXA1	Annexin A1	[12]
P01034	CYTC	Cystatin-C precursor	[12]
P35527	K1C9	Cytokeratin-9	[13]
Q01469	FABPE	Fatty acid-binding protein, epidermal	[12]
P26038	MOES	Moesin	[14]
P62937	PPIA	Peptidyl-prolyl cis-trans-isomerase A	[12]
P01833	PIGR	Polymeric-immunoglobulin receptor	[12]
P05109	S10A8	Protein S100-A8	[12]
P06702	S10A9	Protein S100-A9	[12]
P13646	K1C13	cytoskeletal 13	[13]
P13647	K2C5	cytoskeletal 5	[13]
P06396	GELS	Gelsolin	[12]
P00738	HPT	Haptoglobin	[12]
P16104	H2AX	Histone H2A.x (H2a/x)	[13]
Q16778	H2B2E	Histone H2B type 2-E	[13]
Q06830	PRDX1	Peroxiredoxin-1	[12]
P07737	PROF1	Profilin-1	[11]
P05109	S10A8	Protein S100-A8	[12]
P06702	S10A9	Protein S100-A9	[12]
